# Attitude and Vaccination Status of Healthcare Workers against Hepatitis B Infection in a Teaching Hospital, Ethiopia

**DOI:** 10.1155/2018/6705305

**Published:** 2018-04-02

**Authors:** Mohammed Akibu, Sodere Nurgi, Mesfin Tadese, Wendwesen Dibekulu Tsega

**Affiliations:** ^1^Department of Midwifery, Institute of Medicine and Health Science, Debre Berhan University, Debre Berhan, Ethiopia; ^2^Department of Public Health, Institute of Medicine and Health Science, Debre Berhan University, Debre Berhan, Ethiopia

## Abstract

**Background:**

World Health Organization and Centers for Disease Control and Prevention recommend all health professionals to get vaccinated against hepatitis B virus before they start the clinical attachments during their stay in the medical school. However, only 18–39% of healthcare workers in low- and middle-income countries received the vaccine. Therefore, this study aims to determine the attitude and vaccination status of health professionals working at Adama General Hospital and Medical College.

**Methods:**

An institution-based cross-sectional study was conducted from December 2016 to February 2017 with 403 health professionals working at Adama General Hospital and Medical College. Data were collected using self-administered questionnaire distributed at the participant's work unit and analyzed using SPSS version 20. Multiple logistic regression analysis was conducted to identify factors that affect the complete vaccination status and *p* value < 0.05 was considered statistically significant.

**Result:**

The prevalence of complete vaccination against hepatitis B virus was 25.6%. The most frequently mentioned reasons for not being vaccinated were high cost of the vaccine (41%) and unavailability of the vaccine (36%). More than three-fourths (77.8%) of study participants strongly agreed that hepatitis B is a major public health threat and there was tendency among participants to believe that their profession will put them at increased risk of acquiring the disease (strongly agreed: 75.9%). Attending infection-prevention training [AOR = 2.3; 95% CI, 1.24–6.31], history of exposure to risky behavior [AOR = 5.5; 95% CI, 2.86–9.29], and long years of work experience [AOR = 3.1; 95% CI, 1.98–5.24] were statistically significant with complete vaccination status.

**Conclusion:**

Only one-quarter of health professionals received the recommended full dose of the vaccine. Sustained hepatitis B vaccination programs for healthcare workers need to be established by collaboration of different stakeholders to optimize health professionals' safety against this contagious infection.

## 1. Introduction

Hepatitis B infection has been a major public health threat that affects nearly two billion people worldwide with 350 million chronic cases and more than 2 million deaths every year [1]. The disease is mainly transmitted by percutaneous or mucosal exposure to infected blood or other bodily fluids and numerous forms of human contact have been suggested to transmit hepatitis B virus (HBV): perinatal/mother-to-child, nonsexual, sexual, needle-sharing, and occupational/healthcare-related forms [2]. The disease causes chronic infection, resulting in cirrhosis of the liver, liver cancer, liver failure, and death. Furthermore, extrahepatic lesions can occur in other organs of the body, particularly in the kidney [3].

Since contact with body fluid of an infected person is one of the principal modes of transmission of the causative virus of hepatitis B infection, healthcare workers (HCWs) constitute one of the high-risk groups for this infection because of their repeated exposure [4]. World Health Organization (WHO) estimated that, of the 35 million HCWs worldwide, 3 million experience percutaneous exposure to blood pathogens each year and 2 million of those HCWs are exposed to hepatitis B virus [5–8]. In general, prevalence of HBV infection among healthcare providers is approximately ten times greater than the general population [9]. More importantly, HCWs in developing countries are at serious risk of infection from blood-borne pathogens because of the high prevalence of such pathogens in many poorer regions of the world, especially in endemic areas like Sub-Saharan Africa [10].

Due to the absence of medical treatment that can cure hepatitis B virus (HBV) infection, hepatitis B vaccine is the single most effective and safe strategy for the prevention of the disease if appropriate doses are given during a period of 6 months. The vaccine provides more than 90% effective protection after all doses [11, 12]. As part of occupational safety measures, WHO, Centers for Disease Control and Prevention (CDC), and the Ethiopian Federal Ministry of Health (FMOH) infection-prevention guidelines recommend that all health professionals should be vaccinated against HBV before they started the clinical attachments during their stay in the medical school [13–15]. However, in spite of higher vulnerability among health professionals, the WHO estimate showed that HBV vaccination coverage among HCWs is only 18–39% in low- and middle-income countries compared to 67–79% in developed countries [13].

In Ethiopia, hepatitis B infection cases account for 12% of the hospital admissions and 31% of the mortality in medical wards of Ethiopian hospitals [16]. Specifically, studies conducted on health professionals revealed 9.7% prevalence of hepatitis B surface antigen (HBsAg) [17]. On top of that, some studies also reported lower coverage of hepatitis B vaccination among health professionals [18–21]. Therefore, this study aimed to assess the attitude and vaccination status of health professionals against hepatitis B virus infection and factors associated with complete immunization.

## 2. Methods

### 2.1. Study Design and Setting

The study was conducted at Adama General Hospital and Medical College, Adama, Ethiopia. The city is located 99 km away from Addis Ababa, the capital city of Ethiopia, to the southeast. This teaching hospital has catchment population of about 5 millions, serving as referral hospital for all nearby hospitals and adjacent regions. It has more than 500 healthcare workers providing the service in different units. This is an institution-based cross-sectional study conducted from December 2016 to February 2017.

### 2.2. Study Participants

All health professionals working at Adama General Hospital and Medical College were included into the study irrespective of their working unit and duration of stay to minimize the risk of selection bias.

### 2.3. Variables and Measurement

Complete immunization against hepatitis B virus and attitude of healthcare workers towards hepatitis B infection and its vaccination were the outcome variables measured in the study, whereas various sociodemographic variables (age, profession, working unit, and years of work experience) and occupational variables (training on infection-prevention and history of exposure to risky behavior) were the independent variables. Complete immunization was measured using the following question: “how many doses of the vaccine have you taken?” Taking three or more doses of the vaccine was defined as complete immunization. Attitude was measured on the cumulative score of thirteen questions designed to assess healthcare workers' attitude towards hepatitis B infection and its vaccination. Each attitude question contains ordinal categorical response rated in 5-point Likert scale [i.e., 1 = strongly disagree; 5 = strongly agree] and these questions were adapted from previous literatures [19, 22, 23]. Overall, the scores for each participant were summed and study participants who have responded to ≥60% of attitude questions positively were regarded as having favorable attitude.

### 2.4. Sample Size and Sampling Procedure

Single population proportion formula was used to calculate the sample size given the prevalence of hepatitis B vaccination for healthcare workers of 50% to obtain a relatively larger sample size, confidence level of 95%, and marginal error of 5%. The final sample size was 403 after adjustment for 5% nonrespondent rate. The total sample size was proportionally allocated to each of the working departments in the hospital. The list of health professionals working in each department was obtained from the hospital and simple random sampling technique was employed to select the study subjects from the list.

### 2.5. Data Collection and Quality Control

Data were collected using self-administered questionnaire distributed at the participant's work unit. Data collection was performed by three nursing professionals through distributing and recollecting the questionnaire prepared in English. Pretesting was performed on 5% of the total sample size in other health facilities and a necessary adjustment was made prior to the actual data collection. The questionnaire was also tested for internal consistency (reliability) by Cronbach's Alpha test using Statistical Package for Social Sciences (SPSS) version 20.0. Similarly, content validity was cross-checked by a public health expert. The completeness, consistency, and accuracy of the collected data were examined by principal investigator every day.

### 2.6. Data Processing and Analysis

The data were coded, cleaned, and entered into Epi Info version 7 and it was exported to SPSS version 20 for statistical analysis. First, descriptive statistics were generated followed by binary and multiple logistic regressions to examine the possible association between the determinant and the outcome variable. In this model, *P* value** < 0.05** was used to declare the presence of statistically significant association. The result was reported strictly following STrengthening the Reporting of OBservational Studies in Epidemiology (STROBE) statement (supplementary file (available here)).

## 3. Result

### 3.1. Sociodemographic Characteristics of the Study Participants

A total of 386 participants completed the questionnaire, making a response rate of 97%. More than half (198 (51.2%)) of participants were male and the age of study participants ranged from 21 to 64 with the mean age of 28.45 (±3.2) years. The professional background of respondents was dominated by nurses (203 (52.7%)) followed by medical doctors (52 (13.5%)) (Table 1).

### 3.2. Attitude towards Hepatitis B Infection and Its Vaccination

More than three-fourths (77.8%) of study participants strongly agreed that hepatitis B is a major public health threat. Similarly, more than half (51.2%) of healthcare workers strongly agreed that hepatitis B vaccine should be obligatory to take. There was tendency among participants to believe that their profession will put them at increased risk of acquiring the disease (strongly agreed: 75.9%). Participants also stated that following the infection-prevention guideline has a potential benefit on reducing the chance of contracting hepatitis B infection (strongly agreed and agreed: 85.2%) (Table 2).

### 3.3. History of Occupational Exposure and Perceived Risk of Disease Acquisition

Healthcare workers were asked to rate their perceived risk of acquiring the infection. The respondents reported that they have very high (51 (13.3%)), high (80 (20.7%)), medium (101 (26.1%)), low (142 (36.9%)), and very low (12 (3%)) risk of contracting the disease. Nearly half (182 (47.3%)) of healthcare workers had history of occupational exposure to risky conditions. Unprotected mucocutaneous fluid contact on intact skin (121 (66.7%)), sharp-needle injury (72 (39.6%)), and body fluid splash through body openings (51 (28.1%)) represent the three main forms of exposure. The most common (130 (71.3%)) action taken after the exposure was washing the area of exposure with soap, water, or antiseptic (Table 3).

### 3.4. Vaccination and Postvaccination Testing

Only three in ten (118 (30.4%)) participants had been screened for hepatitis B surface antigen. Regarding the vaccination status of study participants, more than half (223 (57.7%)) of them reported history vaccination at least once. However, less than half (99 (44.5%)) of these participants received the recommended three doses of the vaccine, of which 36 (36.8) tested after the vaccine to check for the vaccine effect and all of them were protected (anti-HB titer > 10 MIU/ml). Among healthcare workers who did not take the vaccine, vaccine unavailability through government channels (36%), high cost of the vaccine for private access (41%), and not giving much concern about this issue (26%) represent the major reasons stated for not being vaccinated (Table 4).

### 3.5. Factors Associated with Vaccination Status

Multivariate analysis of factors affecting the practice of full dose vaccination revealed that previous exposure to occupational risks of hepatitis B infection, years of work experience and infection-prevention training were statistically significant with complete vaccination status. Participants whose years of work experience were ≥5 years had 3 times (AOR = 3.1 (.98–5.24)) greater chance of receiving the vaccine. Likewise, previous history of exposure to occupational risks of hepatitis B infection resulted in 5.5 times (AOR = 5.5 (2.86–9.29)) increased practice of receiving full dose vaccine. Similarly, participants who attended infection-prevention training were 2.3 times (AOR = 2.3 (1.24–6.31)) more likely to take the recommended vaccine dose than their counterparts (Table 5).

## 4. Discussion

Hepatitis B vaccination is one of the most important primary prevention ways of this contagious disease and immunization against this infectious agent provides an optimal protection for individuals at risk [24]. World Health Organization estimated that hepatitis B vaccine's coverage among healthcare providers is 18% in Africa, which represents the least figure [25]. Therefore, this study assessed the coverage of hepatitis B vaccine among healthcare workers of Adama General Hospital and Medical College, Ethiopia.

In this study, it appeared that the proportion of healthcare workers who received hepatitis B vaccine at least once was 57.7%. This finding is in the range of 47%–60% reported in different studies of different areas [20, 26–28]. However, the result is relatively lower compared to the findings reported from Iraq (65.7%), Kuwait (74.4%), India (78%), and Nigeria (91.9%) [29–32]. The complete reason for low vaccine coverage of our survey compared to these studies cannot be completely discernible. However, difference in vaccine accessibility across countries, relatively late addition of hepatitis B vaccine into national immunization program, and certain variability between the sociodemographic characteristics of the study participants might explain this discrepancy. The proportion of healthcare workers who completed the recommended three or more doses of the vaccine constitutes 25.6% of the whole study participants. This figure is lower compared to reports of other studies conducted in Pakistan (57.6%), Malaysia (58.6%), and Libya (72%) [33–35]. This lower rate of complete immunization reflects the need for well effective strategy that enhances increased rate of compliance with recommended vaccine doses. Furthermore, health professionals have to be supported and inspired to check their protection status to make sure whether or not they require additional doses of the vaccine to get protected.

Regarding the attitude of healthcare workers towards hepatitis B infection and its vaccination, the majority of them showed encouraging positive attitude towards the issue. The majority (77.8%) of healthcare workers strongly agreed that hepatitis B is a major public health threat and almost all (96.5%) of them stated that their job puts them at risk of acquiring the disease. Regarding the importance of vaccination, around 75% of healthcare workers agreed that hepatitis B vaccination should be compulsory. These statements are also similarly reported at comparable rate in other studies conducted in Kuwait and Gondar University Hospital [30, 36].

Among healthcare workers who did not receive the vaccine, the most frequently mentioned reason was high cost of the vaccine for private access. Similarly, studies from different areas reported the same finding [20, 22, 37, 38]. Another barrier mentioned was vaccine unavailability, which was reported by 36% of participants. Likewise, this report is in line with the reasons mentioned for vaccine refusal in different articles [19, 28, 39]. This is an input for stakeholders to establish an effective program that focuses on vaccine availability at affordable cost to meet the demand of healthcare workers. According to our study, nearly half (47.3%) of healthcare workers have been exposed to risky situation for hepatitis B infection. Of these, 39.6% reported exposure to sharp-needle injury. A study conducted in Pakistan reported that the percentage of healthcare workers who had experienced at least one sharp injury in a year was 44% and another study conducted in Gondar University Hospital also revealed that 49.2% of healthcare workers had been exposed to occupational risks [36, 40]. This evidence emphasizes the importance of hepatitis B vaccination for this group of people in particular given the extent of their exposure.

This study revealed that healthcare workers who have been exposed to risky conditions of hepatitis B virus had increased chance of receiving complete immunization. This finding is in line with reports from north India, Zambia, and northwest Pakistan [23, 28, 41]. This might be because of increased perceived threat of getting such blood-borne disease after exposure to risky conditions. Years of work experience were another important factor that influenced the complete vaccination status of healthcare workers. Similarly, other studies showed that there was an increased chance to get full vaccination with increasing number of years of work experience [21, 42, 43]. This might be because of the fact that healthcare workers who joined the institutions later might not have benefited from vaccination because of sporadic availability of the vaccine through government channels. Likewise, increased length of work years would result in higher rate of exposure to various risky behaviors, which in turn leads to increased perceived threat of acquiring the disease. Participants who attended infection-prevention training showed increased rate of complete immunization. Studies from Zambia and Nigeria revealed the same finding [23]. This result shows that provision of basic infection-prevention training for all hospital might have a benefit in terms of lifting up the perceived benefit of such preventive strategies among healthcare workers.

## 5. Limitations

Despite extensive efforts that have been made to minimize possible shortcoming of this study, the finding of this survey will be interpreted in the presence of the following inevitable limitations. The cross-sectional nature of the study does not confirm the definitive cause-and-effect relationship. There is also a possibility of admitting recall bias because of the self-reported vaccination status.

## 6. Conclusion

Only a small proportion of healthcare workers have taken the recommended three doses of the vaccine at Adama General Hospital and Medical College. Attending infection-prevention training, work experience, and history of exposure to risky condition were the factors that are statistically significant with the completion of the recommended three doses. High cost of the vaccine for private access and vaccine unavailability were the major barriers identified for hepatitis B vaccination. Sustained HBV vaccination programs for HCWs need to be established by collaboration of different stakeholders. Moreover, the Regional Health Bureau should offer the vaccine to HCWs free of charge by coordinating efforts from other concerned bodies. Similarly, the government should design a national strategy that focuses on vaccinating medical and health science students before they are assigned to their work place.

## Figures and Tables

**Table 1:** Socio-demographic characteristics of health professionals, AHMC, Ethiopia 2017.

Variable	Frequency (*n* = 386)	Percent (%)
Sex		
Male	198	51.2
Female	188	48.8
Age (years)		
20–30	33	16.3
31–40	94	46.3
>40	7	3.4
Marital status		
Unmarried	210	54.4
Married	176	45.6
Religion		
Orthodox	266	69
Protestant	72	18.7
Muslim	35	8.9
Other^a^	13	3.4
Profession		
Nurse	203	52.7
Midwife	44	11.3
Lab technician	25	6.4
General Practitioner	52	13.5
Dental Doctor	14	3.6
Pharmacist	20	5.2
Specialist	28	7.3
Working department		
Inpatient department	92	23.8
Outpatient	114	29.5
Emergency department	36	9.3
Delivery unit	53	13.8
Laboratory	28	7.3
OR department	32	8.3
Dental department	31	8
Work experience		
<5 years	227	58.7
≥5 years	159	41.3
Training on infection prevention		
Yes	224	58
No	162	42

^a^catholic, woke feta, Adventist.

**Table 2:** Attitude of health professional towards hepatitis B infection and its vaccination, AHMC, Ethiopia, in 2017.

Items	Strongly agree	Agree	Neutral	Disagree	Strongly Disagree
HBV is serious public health problem	300 (77.8%)	70 (18.2%)	4 (1.0%)	10 (2.5%)	2 (0.5%)
All patients should be tested for HBV before they receive healthcare	80 (20.7%)	116 (30.1%)	99 (25.6%)	68 (17.7%)	23 (5.9%)
Being a health professional puts you at greatest risk of HBV infection	292 (75.9%)	80 (20.6%)	8 (2%)	4 (1%)	2 (0.5%)
Following infection control guidelines will protect me from being infected with HBV and HCV at work	203 (52.7%)	125 (32.5%)	21 (5.4%)	29 (7.4%)	8 (2,0%)
I deliver the same standard of care to patients with HBV as I do for other patients	46 (11.8%)	148 (38.4%)	32 (8.4%)	101 (26.1%)	59 (15.3%)
It is appropriate not to spend much time when caring HBV-infected patients	91 (23.6%)	109 (28.2%)	46 (11.8%)	72 (18.7%)	68 (17.7%)
A healthcare worker can infect patients with HBV	112 (29.1%)	141 (36.5%)	15 (3.9%)	78 (20.2%)	40 (10.3%)
Health professionals who are hepatitis B virus-positive should not give healthcare services to patients	25 (6.4%)	32 (8.4%)	63 (16.3%)	118 (30.5%)	148 (38.4%)
I do not trust HBV vaccine	17 (4.3%)	45 (11.7%)	54 (14%)	109 (28.3%)	161 (41.7%)
HBV vaccine should be compulsory	198 (51.2%)	91 (23.7%)	25 (6.4%)	64 (16.7%)	8 (2%)
HB vaccine is safe but is expensive	236 (61.1%)	89 (23.2%)	36 (9.4%)	13 (3.4%)	12 (3.0%)
After exposure to contagious flu-id/ material, the vaccine reduces likelihood of being HBV-positive	122 (31.5%)	97 (25.1%)	49 (12.8%)	80 (20.7%)	38 (9.9%)

**Table 3:** Exposure to occupational risk of hepatitis B among health professionals, AHMC, Ethiopia, in 2017.

Variable	Frequency	Percentage
Occupational exposure		
Yes	182	47.3
No	204	52.8
Exposure to sharp injury		
Yes	72	39.6
No	110	60.4
Unprotected mucocutaneous fluid contact on intact skin		
Yes	121	66.7
No	61	33.3
Body fluid contact through body openings		
Yes	51	28.1
No	131	71.9
Measure taken after exposure		
Testing the patient right away	71	39
Washing with soap, water, or antiseptic	130	71.3
Immediate report	80	44.2
Allowing the injury area to bleed	25	14
Wait and test myself	11	6.2

**Table 4:** Vaccination status and reason for not taking the vaccine among health professionals, AHMC, Ethiopia, in 2017.

Variables	Frequency *N*	Percentage (%)
Ever screened for hepatitis B		
Screened	118	30.4
Not screened	268	69.6
Vaccination for hepatitis B		
Vaccinated	223	57.7
Not vaccinated	163	42.3
Vaccination dose		
Once only (incomplete vaccination)	75	33.6
Received two doses (incomplete vaccination)	49	21.9
Three complete doses (fully vaccinated)	99	44.5
Complete vaccination status		
Fully vaccinated	99	44.5
Incomplete vaccination	124	55.4
Have you been tested after full dose?		
Tested for the vaccine effect	36	36.4
Not tested	63	63.8
Test result		
Protected (anti-HB titer > 10 MIU/ml)	36	100
Reason for incomplete vaccination		
Being busy	47	37.9
I feel I am protected	15	12.2
Forget it at all	28	22.6
Waiting for the next dose	34	27.3
Reason for not taking the vaccine		
The vaccine was not available through government channels	59	36
The vaccine is very expensive for private access	67	41
I did not give it too much emphasis	42	26
The side effect would be worse	13	8
The duration of total dose is too long	25	15.4
Others^b^	6	3.7

^b^I do not think I am at risk, I never thought about it, or I have no reason.

**Table 5:** Logistic regression of factors affecting full vaccination status among HCWs who received at least one dose, AMHC, Ethiopia, in 2017.

Variable	Fully vaccinated		COR (95% CI)	AOR (95% CI)
	Yes	No		
Sex				
Male	37	49	1	1
Female	62	75	3.45 (1.82–6.7)	1.06 (0.84–3.62)
Work experience				
**<**5 years	28	74	1	1
≥5 years	71	50	4.8 (2.64–7.44)^*∗*^	3.1 (1.98–5.24 )^*∗*^
Profession				
Nurse	35	45	0.87 (0.67–2.13)	0.22 (0.03–1.45)
Midwife	14	17	1.5 (0.68–2.75)	0.6 (0.07–4.64)
Lab technician	12	13	2.6 (1.36–5.71)^*∗*^	1.72 (0.89–2.42)
General practitioner	16	19	2.1 (0.92–6.37)	0.03 (0.04–2.21)
Pharmacist	5	8	1.65 (0.401–4.62)	0.82 (0.27–3.91)
Dental doctor	6	9	1.39 (0.44–3.64)	1.57 (0.38–6.19)
Specialist	11	13	1	1
Training on IP				
Yes	67	48	3.4 (2.77–8.92)^*∗*^	2.3 (1.24–6.31)^*∗*^
No	32	76	1	1
Work unit				
Inpatient unit	12	25	2.93 (2.91–7.68)	1.07 (0.94–3.75)
Dental department	4	11	0.063 (0.0057–0.84)	0.071 (0.003–1.83)
Emergency unit	13	16	1.4 (0.63–3.18)	1.6 (0.71–4.2)
Delivery unit	23	21	4.64 (1.43–19.7)^*∗*^	2.48 (0.82–7.29)
Laboratory	21	7	5.45 (1.74–9.27)^*∗*^	2.16 (0.018–6.23)
OR department	6	13	1.39 (0.34–3.24)	1.56 (0.40–6.24)
Outpatient unit	20	31	1	1
Exposure history				
Yes	73	38	6.4 (3.43–11.58)	5.5 (2.86–9.29)^*∗*^
No	26	86	1	1

*∗* indicates statistically significant value.

## Data Availability

The original raw data analyzed during the current study is available from the corresponding author and can be presented upon reasonable request.

## Supplementary Materials

Supplementary Materials Supplementary file 1: completed checklist of STrengthening the Reporting of OBservational Studies in Epidemiology (STROBE) for cross-sectional studies. STROBE Statement—Checklist: Attitude and Vaccination status of Health care workers against Hepatitis B infection in a Teaching Hospital, Ethiopia.
